# Pathogenetic and Prognostic Implications of Increased Mitochondrial Content in Multiple Myeloma

**DOI:** 10.3390/cancers13133189

**Published:** 2021-06-25

**Authors:** Yanira Ruiz-Heredia, Alejandra Ortiz-Ruiz, Mehmet K. Samur, Vanesa Garrido, Laura Rufian, Ricardo Sanchez, Pedro Aguilar-Garrido, Santiago Barrio, Miguel A. Martín, Niccolò Bolli, Yu-Tzu Tai, Raphaël Szalat, Mariateresa Fulciniti, Nikhil Munshi, Joaquín Martínez-López, María Linares, Miguel Gallardo

**Affiliations:** 1 H12O-CNIO Hematological Malignancies Clinical Research Unit, CNIO, 28029 Madrid, Spain; yanira_heredia@altumsequencing.com (Y.R.-H.); maortiz@cnio.es (A.O.-R.); gvaneg@hotmail.com (V.G.); ricardo_sanchez@altumsequencing.com (R.S.); paguilar@cnio.es (P.A.-G.); santiago_barrio@altumsequencing.com (S.B.); mlinares@ucm.es (M.L.); mgallardod@cnio.es (M.G.); 2 Hematology Department, Hospital Universitario 12 de Octubre, 28041 Madrid, Spain; lrufian.imas12@h12o.es; 3 Dana-Farber Cancer Institute, 450 Brookline Avenue, M230 Boston, MA 02215, USA; mehmet_samur@dfci.harvard.edu (M.K.S.); yu-tzu_tai@dfci.harvard.edu (Y.-T.T.); raphaelE_Szalat@dfci.harvard.edu (R.S.); mariateresa_fulciniti@dfci.harvard.edu (M.F.); nikhil_munshi@dfci.harvard.edu (N.M.); 4 Instituto de Investigación, Hospital Universitario 12 de Octubre, 28041 Madrid, Spain; mamcasanueva@h12o.es; 5 Centro de Investigación Biomédica en Red de Enfermedades Raras (CIBERER), 28029 Madrid, Spain; 6 Hematology Unit, Fondazione IRCCS Ca’ Granda Ospedale Maggiore Policlinico, 20122 Milan, Italy; niccolo.bolli@unimi.it; 7 Department of Oncology and Hemato-Oncology, University of Milan, 20122 Milan, Italy; 8 Biochemistry and Molecular Biology Department, Pharmacy School, Universidad Complutense, 28040 Madrid, Spain

**Keywords:** multiple myeloma, smoldering MM, mitochondria DNA copy number, NGS

## Abstract

**Simple Summary:**

Monoclonal gammopathies comprise a spectrum of disorders defined by the clonal proliferation of plasma cells and include monoclonal gammopathy of undetermined significance (MGUS), smoldering MM (SMM) and multiple myeloma (MM), which can also evolve from MGUS and SMM. We aimed to analyze the impact of mitochondrial DNA copy number (mtDNACN) and also SNVs and INDELs in frequently mutated mitochondrial-related genes on the disease course of monoclonal gammopathies and MM. We confirmed the increased levels of mtDNA in SMM and MM, their gain as the gammopathy progresses and the similarities in the mitochondrial hallmarks between rapidly-progressing SMM and MM. Our data suggest that mitochondria participate in the malignant transformation of monoclonal gammopathies and contribute to disease progression. Our findings support the clinical importance of mtDNACN evaluation and monitoring to guide clinical decision making in patients with SMM.

**Abstract:**

Many studies over the last 20 years have investigated the role of mitochondrial DNA (mtDNA) alterations in carcinogenesis. However, the status of the mtDNACN in MM and its implication in the pathogenesis of the disease remains unclear. We examined changes in plasma cell mtDNACN across different stages of MM by applying RT-PCR and high-throughput sequencing analysis. We observed a significant increase in the average mtDNACN in myeloma cells compared with healthy plasma cells (157 vs. 40 copies; *p* = 0.02). We also found an increase in mtDNACN in SMM and newly diagnosed MM (NDMM) paired samples and in consecutive relapses in the same patient. Survival analysis revealed the negative impact of a high mtDNACN in progression-free survival in NDMM (*p* = 0.005). Additionally, we confirmed the higher expression of mitochondrial biogenesis regulator genes in myeloma cells than in healthy plasma cells and we detected single nucleotide variants in several genes involved in mtDNA replication. Finally, we found that there was molecular similarity between “rapidly-progressing SMM” and MM regarding mtDNACN. Our data provide evidence that malignant transformation of myeloma cells involves the activation of mitochondrial biogenesis, resulting in increased mtDNA levels, and highlights vulnerabilities and potential therapeutic targets in the treatment of MM. Accordingly, mtDNACN tracking might guide clinical decision-making and management of complex entities such as high-risk SMM.

## 1. Introduction

Mitochondria are bioenergetic and biosynthetic organelles that control crucial biological pathways such as cell growth, proliferation and apoptosis, among others [1,2]. Recently, many studies have investigated the consequences of mtDNA alterations in cancer, demonstrating that cancer cells exhibit multiple alterations in mitochondrial content, structure, function and activity [3,4,5]. Mitochondria appear to be essential for tumor formation and growth [6], and dysfunction in mitochondrial processes affects the electron transport chain and promotes tumor metastasis [7]. Indeed, cancer is characterized by altered energy metabolism involving not only genetic alterations in nuclear DNA, but also mtDNA mutations and changes in mtDNACN [6,8,9]. A recent comprehensive study of the mtDNACN in many cancer types revealed that the majority show changes in mtDNA content when compared with adjacent healthy tissue [9], and also that the mtDNACN is significantly associated with patient survival.

The status of the mtDNACN and its implication in the pathogenesis of cancer is gaining traction [10,11]. These parameters remain, however, relatively unexplored in the context of monoclonal gammopathies and MM, partly because of the inherent difficulties of working with myeloma cells in vitro and partly because of the death of healthy plasma cells in patients with MM. Nonetheless, mtDNA is known to play defining roles during early and late tumor progression in MM [12]. Moreover, mitochondrial biogenesis and activity is often altered in MM due to aberrant gene expression and mutation of mitochondrial-related genes, ultimately contributing to disease progression and relapse, particularly to bortezomib but also to venetoclax [13,14,15,16].

SMM (asymptomatic) is a monoclonal gammopathy with a wide spectrum of progression, and its risk classification has been a major goal in MM research in the last 20 years. Several different models have been tested for predicting progression to active MM—including the Mayo Clinic model, the PETHEMA model and the 20-20-20 model [17]—and novel imaging technologies, such as PET-CT [18], can complement more traditional approaches like fluorescent in situ hybridization and peripheral blood plasma cell quantification [19] to aid in prognostication. Nonetheless, cutting-edge molecular technologies (e.g., next-generation sequencing (NGS) and whole-genome sequencing (WGS)) [20] can offer a more complete molecular profile of SMM, which is crucial to determine the prognosis and classification of SMM and high-risk SMM and to guide clinical decision making in the management of the disease.

Here, we evaluated the mtDNACN state in patients with MM across all stages of the disease, and investigated its impact on patient survival. We also traced the time-evolution of mtDNACN over the course of the disease, which may provide new insight into the role of mitochondria in the progression of MM. Finally, we conducted differential gene expression analysis of mitochondrial biogenesis regulator genes and deep-targeted sequencing of nuclear genes involved in mtDNA replication. Our results provide new evidence supporting the hypothesis that mitochondrial gain-of-function in MM, through increased mitochondrial content, contributes to the transformation process. These observations implicate the mtDNACN as a novel biomarker for MM with prognostic potential, including for risk classification of SMM and rapidly-progressing SMM.

## 2. Materials and Methods

### 2.1. Mitochondrial DNA Copy Number Determination

mtDNACN changes were studied in 4 healthy donors and in 142 patients with MM across different stages of the disease: 17 with MGUS, 20 with SMM, 79 with NDMM and 26 with relapse MM (RRMM). Clinical data from the global dataset are included in Table 1. Analysis was performed using plasma cells enriched from bone marrow aspirates with anti-CD138+ immunomagnetic beads (Miltenyi Biotec, Auburn, CA), with >85% purity in all cases. mtDNACN was quantified by RT-PCR using two TaqMan probes targeting nuclear DNA (RNAseP Control Reaction Kit, Applied Biosystems, Grand Island, NY, USA) and the mitochondrial gene MT-RNR1 (6FAM-5′-TGCCAGCCACCGCG-3′, with 12S forward primers mtF805-New (5′-CCACGGGAAACACGACTGAT-3′) and 12S reverse mtR927 (5′-CTATTGACTT GGTTAATCGTGTGA-3′)) (Thermo-fisher, Waltham, MA, USA) [21].

### 2.2. Deep Targeted Sequencing

Deep targeted sequencing was performed in 13 of the 142 samples with available DNA and mtDNACN quantification (4 MGUS, 1 SMM, 5 NDMM and 3 RRMM). The panel contained the following 12 genes involved in mtDNA replication and frequently mutated in mitochondrial-related diseases: *POLG*, *POLG2*, *MPV17*, *RRM2B*, *DGUOK*, *OPA1*, *TK2*, *TWINKLE*, *SLC25A4*, *SUCLA2*, *SUCLG1* and *MFN2*. Libraries were sequenced on the Ion Proton platform (Life Technologies, ThermoFisher Scientific Inc., Waltham, MA, USA). The average coverage depth was 2000× and the minimum coverage of the detected variants was 20×. Single nucleotide variants were called and annotated using Ion Reporter software (v5.0, Thermo Fisher, Waltham, MA, USA).

### 2.3. Whole-Genome and Whole-Exome Sequencing

Changes in mtDNACN in 10 paired samples from patients with SMM progressing to NDMM were examined by WGS. DNA was extracted from 20 samples of CD138+ myeloma cells purified from bone marrow, and only samples with >90% plasma cells were selected. Paired-end sequencing (100 bp) on the HiSeq2000 sequencing platform (Illumina, San Diego, CA, USA) was performed to an average depth of 38.7×. Sequence files are available at the European Genome-phenome archive under the accession code EGAD00001001898.

Additionally, changes in mtDNACN were evaluated in sequential samples from the same patient, at the time of diagnosis and relapse, by whole-exome sequencing (21 patients, 44 samples). DNA was isolated from CD138+ myeloma cells purified from bone marrow and constitutional control DNA originated from peripheral blood mononuclear cells. Genomic libraries were generated using the Agilent SureSelect Human Exon Kit (Agilent Technologies, Santa Clara, CA, USA; G3362) and were analyzed on the Illumina HiSeq2000 sequencing platform. Paired 75-bp sequences were read with an average depth of 236×. The data have been deposited in the European Genome-phenome Archive repository under the accession code kEGAD00001000339.

mtDNACN was studied from both NGS datasets using FastMitocalc (ultra-fast version of mitoCalc; https://lgsun.irp.nia.nih.gov/hsgu/software/mitoAnalyzer/index.html; accessed on 28 May 2019) [22] and Samtools (version 0.1.19; http://samtools.sourceforge.net; accessed on 10 April 2019) [23] software. Briefly, mtDNACN was calculated after extracting reads aligning to the mitochondrial genome from bam files. Then, copy number variation was calculated by comparing the number of reads aligning to chromosome 10, one of the most stable chromosomes in MM, with the number of reads aligning to an mtDNA reference genome.

(1) $$mtDNA\;copy\;number=\frac{mtDNA\;average\;coverage}{autosomal\;DNA\;average\;coverage\;}\times 2$$

### 2.4. RNA Expression Analysis

We extracted RNA and DNA from purified plasma cells (CD138+) using the AllPrepDNA/RNA Mini Kit (Qiagen, Valencia, CA, USA). Gene expression was studied from extracted RNA processed into cDNA to quantify the levels of Tu translation elongation factor, mitochondrial (*TUFM*) (ThermoFisher Scientific Waltham, MA, USA) and mitochondrial transcription factor A (*TFAM*) (ThermoFisher Scientific). Beta-D-glucuronidase (*GUSB*, ThermoFisher Scientific) was used as a housekeeping gene. This dataset included 47 samples from patients of the Hospital 12 de Octubre (Madrid) that were profiled using TaqMan probes: 9 MGUS, 9 SMM, 18 NDMM and 11 RRMM. qPCR was performed on an ABI PRISM 7900HT instrument (Applied Biosystems, Foster City, CA, USA) with TaqMan gene expression assays (Life Technologies) in triplicate using SDS 2.2 software (Applied Biosystems). The relative level of targets was determined using the comparative Ct method [24].

We also analyzed RNA-seq data from 770 patients with NDMM available at the CoMMpass repository (https://research.themmrf.org and www.themmrf.org; both accessed on 22 April 2020) [25]. The data were generated as part of the publicly available Multiple Myeloma Research Foundation Personalized Medicine Initiatives with the identifier IA14, under “Code availability”. A molecular gene view on the entire dataset was run to study the expression of genes involved in mitochondrial activity: *TUFM* and cytochrome c oxidase II (*COXII*). Kaplan–Meier curves of progression-free survival (PFS) were plotted with balanced stratification of high and low expression of each gene studied.

We also checked differential gene expression of defined pathways at the Molecular Signatures Database (MSigDB), including the mitochondrial respiratory chain pathway (involving 24 genes: *ND1*, *OXA1L, NDUFA2*, *NDUFB6*, *UQCRC1*, *NDUFA9*, *NDUFA6*, *NDUFAB1*, *NDUFA13*, *BCS1L*, *NDUFA1*, *NDUFS7*, *SDHA*, *NNT*, *NDUFS4*, *UQCRH*, *NDUFV1*, *NDUFS8*, *NDUFS3*, *SURF1*, *NDUFS2*, *NDUFS1*, *UQCRB*, *COX15*) and the cytochrome c oxidase activity pathway (13 genes: *COX11*, *COX7A1*, *COX10*, *COX7B*, *COX8A*, *COX4I2*, *COX4I1*, *CYB5A*, *COX7A2L*, *COX5A*, *COX5B*, *SURF1*, *COX15*).

### 2.5. Statistical Analysis

Statistical analysis was performed using GraphPad Software (GraphPad Prism, version 6.0, La Jolla, CA, USA). All data are presented as the mean ± SEM and are representative of three independent experimental replicates (*n* = 3). Normally distributed data were analyzed using the unpaired Student’s *t-*test or one-way analysis of variance (ANOVA) Kruskal–Wallis test. The Mann–Whitney test was performed when data did not pass normality. *p*-values < 0.05 were considered to be statistically significant.

## 3. Results

### 3.1. Patients with Newly Diagnosed MM and Relapsed Patients Have an Increased Mitochondrial DNA Copy Number Associated with Worse Prognosis

**Figure 1:**
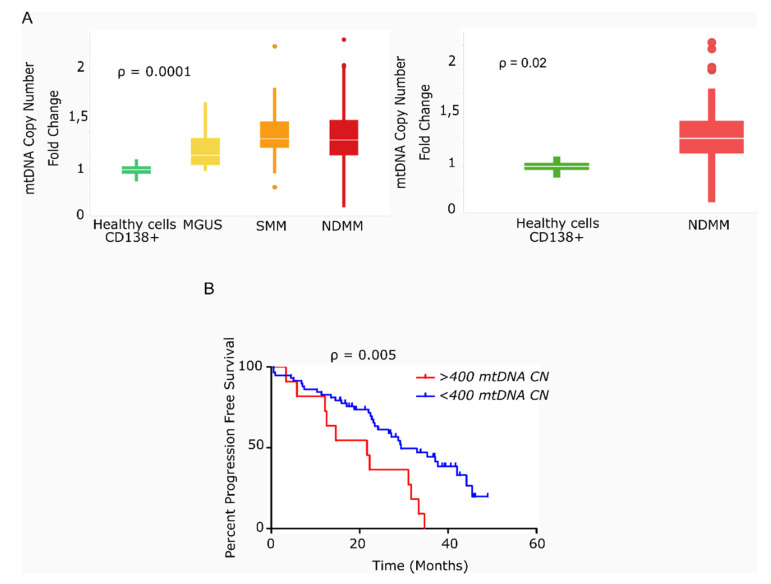
Primary multiple myeloma cells exhibit differences in mitochondrial DNA copy number, which correlates with survival. (**A**) Box-plots of mtDNACN values estimated from next-generation sequencing data (*n* = 142). MM/ healthy donors mtDNACN fold-change (153 vs. 40 copies), *p* = 0.0001 (ANOVA Kruskal–Wallis test). Average mtDNACN of MM cells from patients with NDMM and healthy plasma cells (157 vs. 40 copies), *p* = 0.02 (Mann–Whitney test). Data are presented as mean values ± SD. (**B**) Kaplan–Meier curves of progression-free survival (PFS) in patients with MM. The red curve corresponds to patients with >400 mtDNA copies (*n* = 11). The blue curve corresponds to patients with <400 mtDNA copies (*n* = 58) (*p* = 0.005).

We found that the average mtDNACN was higher in myeloma cells than in healthy plasma cells across the various stages of disease (153 vs. 40 copies; *p* = 0.0001), but no significant differences were observed in mtDNACN between the MGUS, SMM and NDMM subgroups (Figure 1A). A wide range of mtDNACN was detected within each group of patients, particularly for the NDMM subgroup (157 ± 139 copies, Figure 1A). Clinical data from the dataset are included in Table 1. Multiple variables could contribute to this increase in mitochondrial load, including the aberrant expression of mitochondrial genes related to biogenesis or mtDNA replication. Mutational screening of the nuclear genes responsible for mtDNA replication in a small subset of patients (*n* = 12) showed the presence of two mutations in the polymerase gamma gene (*POLG*); a missense variant in a patient with SMM (Ser933Gly, 50% variant read frequency (VRF), 57.1 copies of mtDNA); and a nonsense variant in a patient with RRMM (Glu538* 32% VRF, 214 copies of mtDNA). A third mutation was detected in *POLG2* in a patient with RRMM (Glu67Gln, 37% VRF, 191 mtDNA copies). The former two mutations were predicted to be deleterious by in silico SIFT and PolyPhen analysis (Table 2).

We next evaluated the impact of high mtDNACN on patient outcome in 69 of the 78 patients with NDMM and in available survival data. As shown in Figure 1B, the 11 patients with >400 mtDNA copies (85th percentile) had significantly shorter PFS than the remaining 58 patients (21.6 vs. 29.4 months; hazard ratio (HR) 3.78; 95% confidence interval 1.49–9.63, *p* = 0.005).

### 3.2. Mitochondrial DNA Copy Number Increases with the Progression of MM Disease

**Figure 2:**
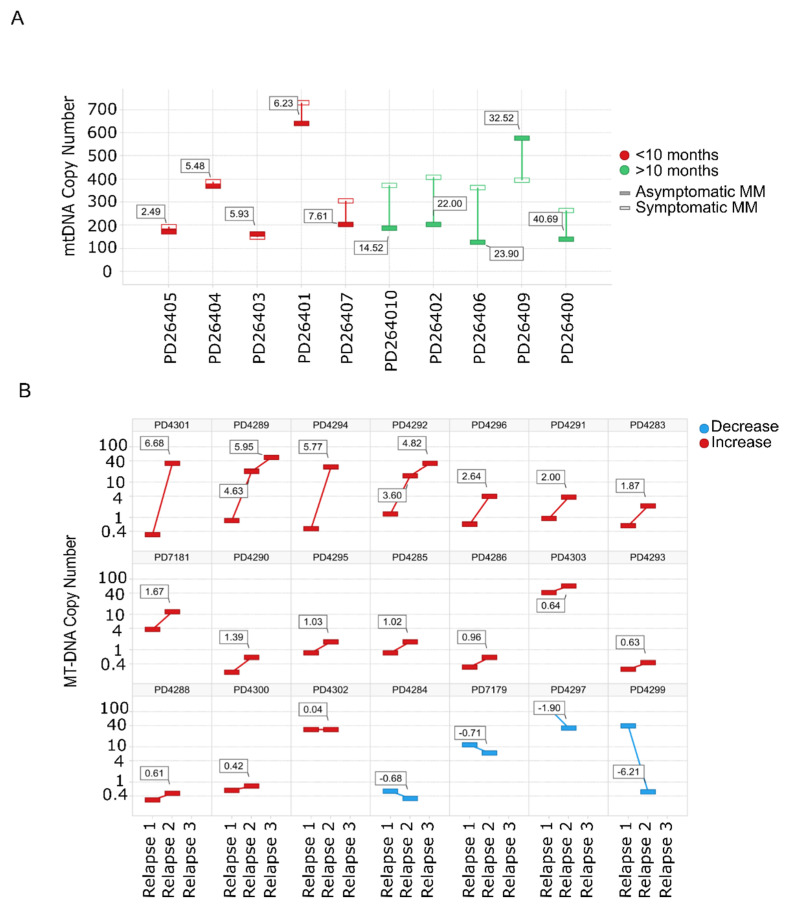
Mitochondrial DNA copy number increases with the progression of the disease. (**A**) mtDNACN calculated from WGS data from paired samples of 10 patients with SMM evolving to MM. Green bars correspond to patients with increased mtDNACN and red bars correspond to decreased mtDNACN, *p* < 0.05 (Mann–Whitney). (**B**) Changes in consecutive relapse patients were assessed from WES data of 21 patients (*n* = 44 samples), *p* < 0.05 (Kruskal–Wallis). Red bars correspond to the increase between the first and second or second and third relapses, whereas blue bars correspond to a decrease.

Evaluation of mtDNACN from WGS data in paired SMM–NDMM samples from the same patient showed that 8/10 patients had an increase in the mtDNACN with progression of the disease. Of note, almost identical mtDNA signatures were found in rapidly-progressing SMM and NDMM (Figure 2A). The same finding was observed for mtDNACN evaluated by whole-exome sequencing (WES) from consecutive relapsed samples from the same patients, with an increase in mtDNACN in 17/21 patients (80%), including those patients with three sequential relapses (Figure 2B).

### 3.3. Mitochondrial Biogenesis Regulator Genes Are Overexpressed in Advance States of MM with Implications for Prognosis

**Figure 3:**
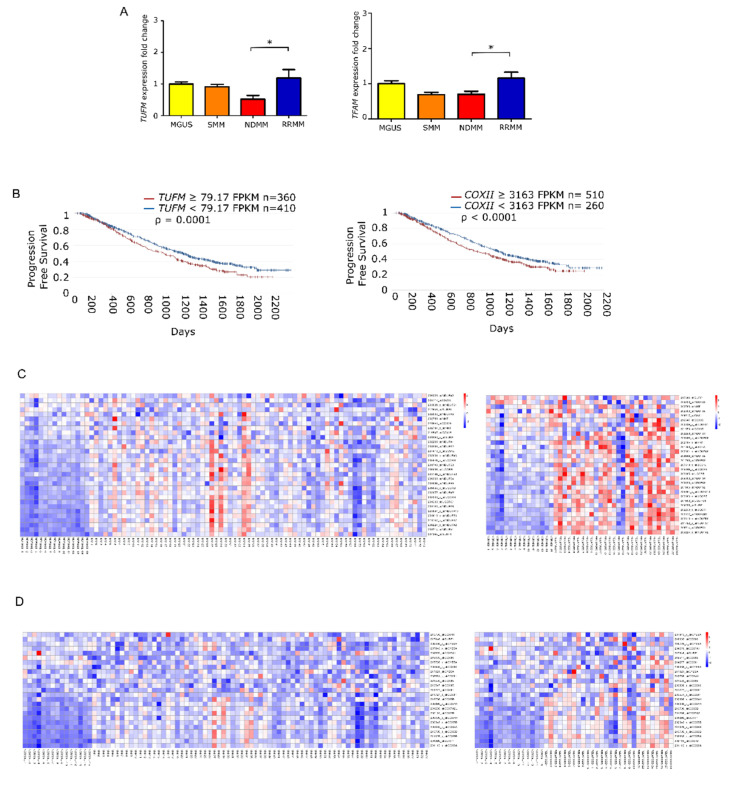
Mitochondrial gene expression is enhanced in late stage multiple myeloma. (**A**) Fold-change in gene expression of *TUFM* and *TFAM* relative to *GUSB* in patients (MGUS *n* = 9; SMM *n* = 9; NDMM *n* = 18; RRMM *n* = 11). Data are presented as mean values ± SEM of technical triplicates. (**B)** High level of mitochondria-related genes identified by RNAseq analysis from the CoMMpass study (IA14); *TUFM* and *COXII* overexpression associate with shorter progression-free survival. (**C**,**D**) Heatmaps of canEvolve database analysis. (**C**) Gene-set enrichment analysis of the mitochondrial respiratory chain pathway; normal vs. NDMM, normal vs. RRMM. **D**. Gene-set enrichment analysis of the cytochrome c oxidase activity pathway; normal vs. NDMM, normal vs. RRMM. * *p* < 0.05.

To elucidate whether the increase in mtDNACN was correlated with the expression of mitochondrial biogenesis regulators, we measured the levels of *TUFM* and *TFAM* by quantitative PCR in the different states of monoclonal gammopathies. The results showed that the expression of *TUFM* and *TFAM* was significantly higher in patients with RRMM than in patients with NDMM (*p* = 0.01 for both genes). Moreover, analysis of variance revealed significant differences between all groups for *TUFM* (*p* = 0.0059) and *TFAM* (*p* = 0.006) (Figure 3A).

Following the same rationale as for mtDNACN, we evaluated whether the overexpression of those genes had any impact on patient PFS. To do this, we used the available RNA-seq data from the CoMMpass database, which corresponded to 770 patients with NDMM. We found that PFS was significantly shorter in those patients with overexpression in genes involved in mitochondrial biogenesis, including *TUFM* (HR 1.27) and *COXII* (HR 1.28) (Figure 3B). Additionally, we assessed the differential gene expression of MSigDB-defined pathways. The mitochondrial respiratory chain pathway (involving 31 genes) showed statistical differences between patients with NDMM and RRMM compared with healthy donors. The cytochrome c oxidase activity pathway (13 genes) showed the same trend (Figure 3C).

## 4. Discussion

Here, we report that elevated mtDNACN, which reflects the abundance of mitochondria, is an additional feature of MM. Our data provide evidence that the malignant transformation of myeloma cells is coupled to the activation of mitochondrial biogenesis, resulting in increased mtDNA levels [3,4,5] and mtDNACN changes [6,8,9]. Indeed, mtDNA can define early and late tumor progression in MM [12].

Monoclonal gammopathies, including MGUS, SMM and MM, are a group of disorders that share a common feature of clonal plasma cell proliferation. MGUS and SMM can evolve to MM through molecular mechanisms that are not fully understood. The biology of myelomagenesis establishes the basis of post-germinal center B-cell evolution to MGUS, SMM and, ultimately, MM. The most accepted theory postulates the existence of primary genetic events, such as IgH translocations, hyperdiploidy and cyclin D dysregulation [26], that trigger monoclonal gammopathies. There are also secondary genetic events that seem to be acquired for the progression of SMM to MM, including mutations in NRAS, KRAS, BRAF or NFKB pathways, loss-of-function of p53, PTEN or RB, and other genetic events, such as secondary translocations, miRNA changes and *MYC* upregulation [27]. All of these chromosomal/genetic abnormalities are linked to multiple phenotypic features, including osteoclast activation and osteoblast inhibition [28], homing of MM cells to the bone marrow niche and immune evasion.

Mitochondrial biogenesis is regulated by myriad molecules, with several implicated in MM disease. For instance, p53 can influence mitochondrial function and governs apoptosis [29]. Also, Myc is implicated in both mitochondrial biogenesis and MM, being commonly overexpressed and associated with its progression. *MYC* regulates several mitochondrial genes such as *COX*, *POLG* and *TFAM*, which are also altered in patients along MM progression [13,30,31]. We show that elevation of mtDNACN along MM progression correlates with increased expression of genes involved in mitochondrial biology (e.g., *TUFM* and *TFAM*). Mitochondrial biogenesis and activity is altered by aberrant gene expression and mutation of mitochondrial-related genes in MM disease and progression, and contributes to relapse [13,14,15,16]. Indeed, we found the presence of pathogenic mutations in genes involved in mtDNA replication, such as *POLG* and *POLG2*, which dysregulate mitochondrial biogenesis and could contribute to mtDNA increases [32].

Mitochondrial biogenesis is increased as a consequence of molecular dysregulation of myelomagenesis events, with a concomitant increase of mtDNACN. We recently described an increase in mitochondrial activity during the progression of monoclonal gammopathies [13], and established mitochondria as a novel therapeutic target of the disease to override the resistance to frontline treatments [13]. Ortiz-Ruiz et al. described the gain of mitochondrial content and its consequent increase of OXPHOS due to *MYC* dysregulation that leads to an increase of mitochondrial biogenesis in vitro, which was also confirmed in the current article in clinical patients. Lack of response to treatments triggered by mitochondrial dysregulation could explain the poor prognosis of patients with an increase in mtDNACN or in mitochondrial biogenesis gene expression [13]. Thus, Ortiz-Ruiz et al. described mechanistically some of the findings of the clinical data showed in the current article.

Within non-myeloma monoclonal gammopathies, SMM is especially challenging in terms of clinical management due to the wide variability in its progression and its risk classification is an important clinical aspiration (e.g., the Mayo Clinic model [17]). While critical advances have been made in the knowledge of SMM, it remains crucial to fully understand and determine the prognosis and classification of SMM and high-risk SMM in order to guide decision making in the management of the disease. Our findings suggest the use of mtDNACN monitoring in monoclonal gammopathies, especially in SMM. Although risk factors have now been defined by the International Myeloma Working Group, controversy exists around the definition of high-risk SMM and its management. Our data support the implementation of mtDNACN analysis, a technique available in the clinic for multiple mitochondrial diseases, in monoclonal gammopathies, especially in SMM and MM.

## 5. Conclusions

Our results demonstrate that elevation of mtDNA content occurs in monoclonal gammopathies (MGUS and SMM) and MM as the disease progresses, and it is notably enhanced in those patients who relapse. Increases in mtDNACN might be linked to aberrant genetic events, such as *POLG* mutations or *MYC* upregulation, as we recently described [13], or to other genetic events triggering an increase of mitochondrial gene expression (e.g., *TFAM*, *TUFM*), load and activity, contributing to poor prognosis and treatment resistance. Additionally, our data provide novel insights into patients with rapidly-progressing SMM and those who relapse, and the potential value of mtDNACN status and evolution for classifying and supporting clinical decisions for these patients. Further analysis in mitochondrial content and activity and confirmative studies with additional samples should be pursued to unravel the role of mitochondria in monoclonal gammopathies.

## Figures and Tables

**Table 1:** Clinical characteristics of patients included in the study (*n* = 142).

		MGUS (*n* = 17)	SMM (*n* = 20)	NDMM (*n* = 79) ^a^	RRMM (*n* = 26)
Median age (range), years		71 (48–87)	78.5 (54–87)	72 (62–85)	67.5 (38–87)
Sex (%)	Male	58.8	45	52.9	53.8
	Female	41.2	55	47.1	46.2
PC BM, average % (range)		6.8 (2–17)	23.2 (10–55)	43.9 (10–84)	39.2 (4–88)
PPC MFC %, average (range)		1 (0.1–2.9)	6.1 (0.98–24)	11.2 (0.2–30)	14.8 (0.01–79)
Type of Ig heavy chain (serum)	Non-detected	5.9	0	0	0
	IgG	52.9	60	64.7	69.2
	IgA	35.3	40	23.5	15.4
	IgM	5.9	0	5.9	15.4
	IgD	0	0	0	0
	Biclonal	0	0	0	0
Type of Ig light chain (serum)	Non-detected	5.9	0	0	3.8
	Kappa	52.9	55	76.5	61.5
	Lambda	41.2	45	23.5	30.8
	Biclonal	0	0	0	3.8
Serum M-spike, ≥3 gr/dL (%)		0	10	82.4	23.1
Urine M-spike, detected (%)		20	29.4	70.6	53.8
Kappa (%)	≥19.4 mg/L	57.1	66.7	100	66.7
Lambda (%)	≥26.3 mg/L	50	41.7	33.3	33.3
Free kappa/lambda ratio (%)	<0.26 mg/L	14.3	23.1	0	26.7
	≥0.26 < 1.65 mg/L	42.9	38.5	22.2	6.7
	≥1.65 mg/L	42.9	38.5	77.8	66.7
Creatinine (%)	≥1.3 mg/dL	29.4	25	47.1	19.2
Serum calcium (%)	≥11 mg/dL	0	0	11.1	11.5
LDH (%)	≥225 U/I	5.9	14	11.8	38.5
Albúmina (%)	≤3 g/dL	0	5	11.8	3.8
Immunoparesis, yes (%)		18.8	20	23.5	80.8
Refractory, yes (%)		NA	NA	5.9	40
Type of prior treatment (%)	With PI	NA	0	5.9	42.3
	Without PI	NA	100	94.1	57.7
Type of following treatment (%)	With PI	NA	25	88.2	42.3
	Without PI	NA	75	11.8	57.7
Best response categories * (%)	VGPR	0	0	17.6	0
	PR	0	30	58.8	34.6
	CR	0	0	23.5	19.2
	CR MRD+	0	0	0	0
	CR MRD-	0	0	0	0
	SD	0	5	0	34.6
	NA	100	65	0	11.5
Performed analysis (methods)	Samples, *n*	26	39	107	81
	WGS	0	10	10	0
	WES	0	0	0	44
	mtDNA CN	17	20	79	26
	Gene expression	9	9	18	11

Percentage frequencies of clinical parameters from patients. Abbreviations: BM, bone marrow; CR, complete response; Ig, immunoglobulin; LDH, lactate dehydrogenase; NDMM, newly-diagnosed multiple myeloma; MFC: multiparametric flow cytometry; MGUS, monoclonal gammopathy of uncertain significance; MRD-, minimal residual disease negative; MRD+, minimal residual disease positive; mtDNACN, mitochondrial DNA copy number; NA, not applicable; PC, plasma cell; PI, proteasome inhibitors; PPC: polyclonal plasma cell; PR, partial response; RRMM, relapse multiple myeloma; SMM, smoldering multiple myeloma; SD, stable disease; VGPR, very good partial response; WES, whole-exome sequencing; WGS, whole-genome sequencing. * Objective response was assessed at the start of each cycle and confirmed by the physician using IMWG criteria. ^a^ MM patients comprised newly-diagnosed and follow-up patients.

**Table 2:** Mutations responsible for mtDNA replication in a small subset of patients (*n* = 12).

Coordinates	Codons	Protein	Substitution	Region	dbSNP ID	SNP Type	Mutated Sample	Prediction
15,89864181,1,T/C	AGT-gGT	POLG	S933G	EXON CDS	Novel	Nonsynonymous	SMM	DAMAGING
15,89869943,1,C/A	GAG-tAG	POLG	E538X	EXON CDS	rs138413938:A	Nonsynonymous	RRMM	Damaging due to stop
17,62492888,1,C/G	GAG-cAG	POLG2	E67Q	EXON CDS	Novel	Nonsynonymous	RRMM	TOLERATED

## Data Availability

The RNA-seq data from the CoMMpass that were analyzed in this study are publicly available through the Multiple Myeloma Genomics Initiative (https://research.themmrf.org; accessed on 22 April 2020) with the identifier IA14.
